# Barriers and Facilitators Concerning Involuntary Oral Care for Individuals with Dementia: A Qualitative Study

**DOI:** 10.3390/ijerph22030460

**Published:** 2025-03-20

**Authors:** Maud Jonker, Coos Engelsma, David J. Manton, Anita Visser

**Affiliations:** 1 Department of Gerodontology, Center for Dentistry and Oral Hygiene, University Medical Center Groningen, University of Groningen, 9713 AV Groningen, The Netherlands; a.visser@umcg.nl; 2 Medical Ethics and Decision Making, Department of Ethics, Center for Dentistry and Oral Hygiene, University Medical Center Groningen, University of Groningen, 9713 AV Groningen, The Netherlands; j.engelsma@umcg.nl; 3 Department of Cariology, Center for Dentistry and Oral Hygiene, University Medical Center Groningen, University of Groningen, 9713 AV Groningen, The Netherlands; d.j.manton@umcg.nl; 4 Department of Paediatric Dentistry, Academic Centre for Dentistry Amsterdam (ACTA), Vrije Universiteit Amsterdam and University of Amsterdam, 1081 LA Amsterdam, The Netherlands; 5 Department of Gerodontology, Faculty for Dentistry, Radboud University Medical Center, Radboud University Nijmegen, 6525 EX Nijmegen, The Netherlands

**Keywords:** involuntary oral care, dementia, oral health care, care-resistant behavior, elderly, nursing homes

## Abstract

Many individuals with dementia show care-resistant behavior toward oral care, while care providers are often reluctant to provide it involuntarily, risking negative health outcomes. This study aims to identify the barriers and facilitators of providing involuntary oral care for individuals with dementia who show care-resistant behavior. In total, 32 semi-structured one-on-one interviews with healthcare providers were conducted. Through the interviews, multiple barriers and facilitators were identified and divided into four main themes, each containing multiple sub-themes: (1) communication (between dental and non-dental care providers, and amongst non-dental care providers themselves), (2) logistics (materials, transportation, and staff and time), (3) knowledge (training, awareness of oral health problems, and assessment of severity of oral health problems), and (4) oral care provision (psychology care providers, attitude concerning involuntary oral care, ethical and legal considerations, and sedation). Our study shed more light on the barriers and facilitators regarding involuntary oral care provision to older individuals with dementia. Multiple recommendations were provided, including designating nurses to help monitor oral health, involving dental professionals in multidisciplinary team meetings, discussing a shift in attitude concerning oral care, providing clear guidelines and protocols for sedation and daily oral care provision, and performing more research into involuntary oral care.

## 1. Introduction

Currently, more than 55 million individuals are living with dementia worldwide, and this number is expected to increase by 10 million individuals each year [1]. Dementia is the collective term for more than fifty different brain disorders, including Alzheimer’s disease, Lewy body dementia, and vascular dementia [2]. Individuals with dementia experience a decline in cognitive function, which ultimately interferes with their ability to perform activities of daily living (ADLs), such as clothing, bathing, and tooth brushing [3]. As a result, individuals with dementia become increasingly dependent on others for care.

This dependency constitutes a significant challenge, especially in oral health care [4,5]. Presently, an increasing number of older individuals with dementia still retain (some of) their own teeth but are often unable to take care of them [4,6]. Consequently, their oral health deteriorates, which can cause pain and infections [7]. Poor oral health is also associated with multiple general health conditions, such as endocarditis, pneumonia, and diabetic control [8]. Although assistance in providing oral care is important, many individuals with dementia show resistance toward oral care [9].

Providing oral care despite care-resistant behavior is considered legally to be involuntary oral care [10]. Involuntary oral care should only be used as a last resort to prevent serious harm because involuntary care can cause the patient to become agitated, depressed, humiliated, and distressed [11]. Additionally, it may also affect care providers, causing, for example, moral distress [11]. Therefore, many countries have laws to regulate the provision of involuntary oral care. In Norway, it is regulated by the Health and Care Services Act; in the Netherlands, it is regulated by the Care and Coercion Act (CCA, Dutch: Wet zorg en dwang) [10,12].

Typically, such laws regulate involuntary oral care by requiring care providers contemplating involuntary treatment to follow specific procedures before providing care. For instance, the Dutch Care and Coercion Act states that the provision of involuntary oral care is only allowed to prevent (the risk of) serious harm [10]. Prior to the provision of involuntary care, specific steps must be undertaken. The first step is to search for voluntary strategies and interventions to provide oral care, such as using distraction techniques (for instance, music or a favorite TV program), with the help of a loved one, and attempting care at different times. When all these voluntary strategies are unsuccessful, and the individual with dementia remains resistant, care providers face a decision of whether to provide oral care against the expressed will of the individual with dementia, or to cease oral care, thereby risking potential negative health outcomes. This decision should be discussed at a multidisciplinary meeting (MDM) where different care providers determine whether the provision of involuntary oral care is the only remaining option, and whether the intended involuntary oral care fits the following three criteria: (1) proportionality: the care is in reasonable proportion to its purpose; (2) subsidiarity: no other, less drastic care is possible to reach the purpose; and (3) effectivity: the care achieves the intended purpose and does not last longer than necessary [10].

A recent questionnaire-based survey of 309 Dutch healthcare providers reported that most care providers do acknowledge that oral health problems of individuals with dementia are harmful, but that many are still reluctant to provide involuntary oral care [13]. In order to comprehend the reluctance of care providers to offer involuntary oral care to individuals with dementia, it is important to identify the barriers to and facilitators of providing involuntary oral care. A literature review by Hoben et al. [14] and a mixed methods study by Weening-Verbree et al. [15] already described the barriers and facilitators of oral health care to nursing home residents in general, including care-resistant behavior [14,15]. However, neither addressed the barriers and facilitators that arise when providing involuntary oral care to individuals with dementia who show care-resistant behavior, nor did they take current laws regulating involuntary care into account. 

More research is crucial to understand the perspectives of all parties involved in decisions on whether to provide involuntary oral care to individuals with dementia who show care-resistant behavior. With the information thereby gained, tailored interventions and guidelines can be developed to facilitate the provision of involuntary oral care as a last resort. Hence, the aim of the present study was to identify the barriers and facilitators of care providers regarding involuntary oral care to older individuals with dementia who show care-resistant behavior.

## 2. Materials and Methods

### 2.1. Study Design, Sample Size, Setting, and Participants

To identify the barriers and facilitators concerning involuntary oral care provision to older individuals with dementia who show care-resistant behavior, an inductive qualitative research design was used. In more detail, semi-structured interviews were conducted between December 2023 and October 2024 with professional healthcare providers involved in oral care for individuals with dementia in the Netherlands. A facilitator was defined as anything contributing to the provision of involuntary oral care; a barrier was defined as anything hindering the provision of involuntary oral care.

The inclusion criterion for participants was being a registered healthcare provider involved in decision making about, or provision of, involuntary oral care to individuals with dementia who show care-resistant behavior in the Netherlands.

The sample size was based on data saturation; therefore, the study responses were monitored by two researchers (M.J. and C.E.) to identify when data saturation occurred. This occurred when M.J. and C.E. agreed that three consecutive interviews did not provide any new insights.

### 2.2. Recruitment Procedure

The recruitment procedure was based on creating a maximum variation sample size, using purposive sampling. Therefore, individuals of different ages, sexes, provinces in the Netherlands, and different occupations were sought to participate in this study. Potential participants were approached through several methods, including the authors’ network, participant lists of a geriatric sedation education program, and targeted outreach via social media to specific healthcare providers.

### 2.3. Participant Information and Informed Consent

Before the interview, participants received study information, including an informed consent form, by e-mail. Hereby, participants were informed that the interview would be recorded and that the results would be made completely anonymous. Next, the interviews were conducted in Dutch via video calls (Microsoft Teams; Microsoft Corp., Redmond, WA, USA) by one researcher (M.J.), a dentist with specific training in qualitative research in healthcare. Before the interview recordings started, participant consent was confirmed. During the interview, an interview guide (Table 1) was used as a flexible guideline to allow expansion on certain topics or comments by the participant. Afterward, all interviews were transcribed verbatim by one researcher (M.J.).

Prior to data collection, pilot interviews were conducted to test the interview guide. During the data collection, the first two interviews of the study were discussed between three researchers (M.J., C.E., and A.V.) to further evaluate the interview guide and the interview technique of the interviewer (M.J.).

### 2.4. Data Analysis

The results were qualitatively analyzed by applying Braun and Clarke’s thematic analysis [16]. After the interviews were transcribed, they were uploaded to Atlas.ti (version 9.13.2, Scientific Software Development GmbH, Berlin, Germany), which was used to code the transcripts. Two researchers (M.J. and C.E.) familiarized themselves with the transcripts, coded the transcripts, and developed categories independently. Subsequently, sub-themes and main themes were created and reviewed together. Consensus was reached through discussion.

## 3. Results

In total, 32 semi-structured interviews were conducted with care providers throughout the Netherlands. Interviews were conducted with eight geriatric-focused dentists, three geriatric-focused dental hygienists, six carers, seven nurses, three geriatric specialists, two dementia case managers, and three general medical practitioners. The average age of the participants was 47 years (range 27–79 years); and most were female (N = 28, 87.5%). The interviews lasted between 19 and 46 min. Table 2 provides a summary of the participants’ characteristics.

### 3.1. Barriers and Facilitators

**Figure 1:**
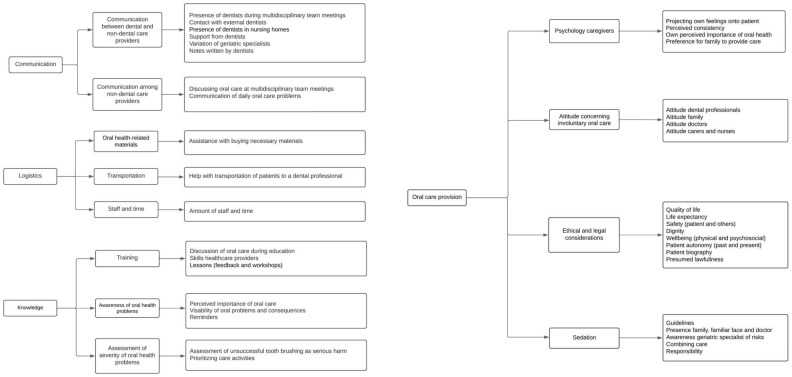
Overview identified barriers and facilitators concerning involuntary oral care provision divided into main themes, sub-themes, and categories.

Multiple barriers and facilitators concerning involuntary oral care provision to older individuals with dementia were identified, and categorized into four main themes: communication, logistics, knowledge, and oral care provision. Each of these themes contains multiple sub-themes, which are explained and illustrated below using quotes extracted from the interviews. The quotes have been translated into English by an experienced Dutch English speaker, as the interviews were performed in Dutch. The quotes were back-translated to ensure their original meaning. The translated quotes were minimally refined to enhance readability, without altering the participant’s meaning. An overview of the main themes, sub-themes, and categories is shown in Figure 1.

#### 3.1.1. Communication

The identified barriers and facilitators regarding communication were categorized into two sub-themes: (A) communication between dental and non-dental care providers, and (B) communication amongst non-dental care providers themselves.

(A) *Communication between dental and non-dental care providers*

Communication between dental and non-dental care providers was compromised by the fact that dentists were often absent during multidisciplinary team meetings (MDMs), mostly due to practical and financial reasons. Many nursing homes lacked an affiliated dentist, and when one was available, they often worked part-time, making it hard to arrange an MDM that they could attend. In addition, dentists were often not invited to participate in an MDM.

*“We are never invited to the multidisciplinary meetings, for example. We are kind of a separate entity that is a bit attached.”*

(Dentist, Interview 6)

Participants described the presence of a dentist during MDMs about patients with (part of) their own dentition or implants as beneficial because oral care would be discussed during these MDMs, and other care professionals would gain (more) insight into the oral health of their patients. Besides providing information to non-dental care providers, the dental professional could gain insight into all other (medical) care aspects of the patient, which could be beneficial for their oral health treatment plan.

Another factor benefiting the communication between dental and non-dental care providers was the presence of dental care professionals affiliated with the nursing home. Caregivers could easily consult with these dental care professionals regarding problems encountered in administering oral care.

*“The question actually came from the care staff themselves, saying that brushing wasn’t going well. Do you have any tips or advice? So, then I came by, and I gave those tips.”*

(Dental Hygienist, Interview 2)

Communication between dental and non-dental care providers was compromised by the fact that several carers and nurses did not have direct contact with external dental care professionals. In such cases, communication with the dental care professional was conducted through the patient’s family, which sometimes led to confusion or uncertainty.

*“You don’t have direct contact with the dentist. The family goes, and then it’s okay, someone has received special toothpaste. Then I just note that in the care plan for brushing with the special toothpaste. But otherwise, it’s always a matter of waiting to see, if someone has been to the dentist, what will we hear?”*

(Carer, Interview 4)

Another factor that can compromise communication between dentists and non-dental care providers, even when the dentists are affiliated, is the lack of personalized notes by dentists. Often, dentists made standard notes that were not specific to a patient after a dental check-up. As a result, carers and nurses were sometimes unaware of relevant oral circumstances.

*“…the dentist gave standard basic notes from their dental check-ups, and they were not personalized for the residents. They don’t give information if the lady needs assistance with oral care, whether she can do it herself or not, or if she needs to be flossed or not and how often, etcetera. And in care plans, it often didn’t even state whether someone has a prosthesis, a partial prosthesis, or no prosthesis.”
*

(Nurse, Interview 4)

The communication between dentists and geriatric specialists also involved challenges. For example, in some nursing homes, there was a high turnover of geriatric specialists. As a result, dentists often had to keep rebuilding the bond with the geriatric specialist as well as providing them with oral health knowledge. This variation in geriatric specialists created a distance between the dentist and the geriatric specialist.

(B) *Communication between non-dental care providers themselves*

Focusing on the communication between non-dental care providers themselves, it was reported that oral care was discussed minimally during the MDMs. Also, one geriatric specialist noted that they scarcely heard about daily oral health issues from nurses and carers. Geriatric specialists only became involved when there was an oral health problem (for example, pain or reduced eating behavior) and they were not contacted when patients showed resistance toward tooth brushing.

*“…but maybe I don’t hear directly from what is happening in practice. We are similar to general practitioners, of course. We take action when there is a problem.”*

(Geriatric specialist, Interview 1)

The geriatric specialist’s being uninformed about daily oral care problems coheres with carers reportedly not communicating about problems regarding daily oral care with colleagues. However, if unsuccessful tooth brushing was not reported, other care providers could be unaware of any problems.

*“I have never mentioned in the report whether I have cleaned someone’s teeth or not.”*

(Carer, Interview 1)

*“…the geriatric specialist is consulted for pain complaints. But regarding dental maintenance, we have never really discussed anything about this with the doctor.”*

(Nurse, Interview 5)

#### 3.1.2. Logistics

Several barriers and facilitators were related to logistics. These were divided into three sub-themes: (A) oral health-related materials, (B) transportation, and (C) staff and time.

(A) *Oral-health-related materials*

With regards to oral-health-related materials, in some nursing homes, the residents needed to provide their own toothbrush and toothpaste. However, some residents had no family caregiver or someone close to them and, therefore, did not receive these necessary materials.

(B) *Transportation*

Transportation to a dental clinic can be difficult, as many older individuals with dementia cannot transport independently or show care-resistant behavior toward transportation. Transportation to the dental clinic is necessary when a dentist does not visit the nursing home or due to the complexity of the dental treatment.

*“Most people with dementia no longer drive. Sometimes, if the children live far away or they have a very small social network, and they need to go somewhere, that is often very complicated. People can no longer take a taxi by themselves because they do not understand how to call a taxi. And it is always nice when someone accompanies them to the dentist.”*

(Dementia case manager, Interview 2)

(C) *Staff and time*

Thirdly, whilst a lack of staff and time was a reported barrier, the presence of enough (or more) staff and time would be a facilitator for the provision of (involuntary) oral care.

*“I think that if we had the manpower, the time, and the budgets and things like that, we would address everything all at once right away. But I also know that from management’s perspective, that’s just not possible, and decisions are made about which group is going to be prioritized. We’re simply dealing with the heavy workload in healthcare. We also face illness and absenteeism.”*

(Nurse, Interview 3)

#### 3.1.3. Knowledge

Barriers and facilitators regarding knowledge were categorized into three sub-themes: (A) training, (B) awareness of oral health problems, and (C) assessment of severity of oral health problems.

(A) *Training*

Many nurses and carers stated that during their education the subject of oral care was barely discussed or not mentioned at all. When addressed, the educational program focused briefly on practical actions, such as toothbrushing, and did not address the importance of oral health and the consequences of poor oral health.

*“In my training as a carer, we had to brush each other’s teeth once, and that was it.”*

(Carer, Interview 5)

Given their limited education about oral health care, a dental hygienist questioned whether one could expect carers and nurses to provide good oral health care to individuals who resist care, especially when they do not know how to manage routine situations.

*“…because essentially you are asking caregivers to start climbing Mount Everest, while they can’t even get up the Kardinge hill [30 m high].”*

(Dental hygienist, Interview 1)

Moreover, nurses and carers expressed a desire to receive tips and tricks from a dental professional on how to administer oral care when an individual resists, and information on the consequences of poor oral health.

*“Yes, and I think it could be improved if we were given more tools on how to approach it—tips and tricks, so to speak, on what you can do to provide the best possible oral care. […] A clinical lesson or something by a dentist, maybe with some photos as well. How teeth can end up looking if not cared for, and the risks and consequences—people really need to become more aware of this.”*

(Nurse, Interview 1)

Similar to the education of nurses and carers, oral health was also discussed minimally in the education of geriatric specialists.

*“It may also be a matter of knowledge about oral care. That is quite minimal. Looking back at my training to become a geriatric specialist, it [oral health] was only a very small part of the curriculum.”*

(Geriatric specialist, Interview 1)

Consequently, a dentist stated that they gave information to the geriatric specialist concerning oral health, and that they invited them to participate in a day of job shadowing to gain more insight into oral health challenges and treatments.

*“For 28 years, I invited all new geriatric specialists who joined my organization to come and observe for a morning. Not just observe—I put a white coat on them. I had them assisting me with suctioning and showed them what was present in the mouth, ideally during an extraction. Really being able to see and experience the patient, that you also want to suture. Just the whole process, from A to Z.”*

(Dentist, Interview 8)

(B) *Awareness of oral health problems*

Regarding awareness of oral health problems, several non-dental care providers conceived oral care as relatively unimportant. For instance, multiple non-dental care providers mentioned that oral care itself was often forgotten, and they did not always consider pain resulting from oral problems as a potential cause of a patient’s behavioral changes.

*“I must say that oral care is often forgotten. That’s really how it is. A neglected stepchild who doesn’t receive enough attention.”*

(Case manager dementia, Interview 1)

*“…and if there is any change in behavior, I think we don’t always consider whether pain from the jaw or dental elements might also play a role, and I must admit, we don’t have a very clear picture of that.”*

(Geriatric specialist, Interview 1)

A lack of awareness of oral health problems could be caused by non-dental care providers physically not seeing oral health problems and their consequences. On the one hand, care providers did not see oral health problems because they are ‘behind closed lips’. On the other hand, not providing oral care (for example, not brushing someone’s teeth) does not directly lead to consequences (such as pain), making it more difficult for non-dental care providers to realize the importance of it.

*“But I think that, in the end, they [non-dental care providers] don’t see the importance of it. Look, if you have a wound and it’s not properly cared for, people get sick from it, and then it’s very clear that someone can die from sepsis. But that is not at all so clear when it comes to dental care. […] A beard, you see that, right? If someone is unshaven, if someone has greasy hair, you see that, but I don’t see a filthy mouth, and people just don’t see it.”*

(Geriatric specialist, Interview 2)

To raise more awareness of oral health problems, multiple participants described the importance of repetition. For example, certain nursing homes implemented themed months, where they focused on a specific topic, such as oral care. As a result, caregivers were reminded of oral care and its importance.

*“‘Well, if I have to brush involuntarily, that’s not allowed, so I don’t do it, right?’ They [other carers] take it [not performing oral care] very lightly, until we have another themed month or something about oral care. Then they think: ‘oh yes, oral care, let me look into that again’.”*

(Nurse, Interview 2)

(C) *Assessment of severity of oral health problems*

Focusing on the third sub-theme, assessment of the severity of oral health problems, some participants indicated they often did not regard the unsuccessful provision of tooth brushing a serious harm for the patient:

*“A serious harm? Honestly not, but it is of course important that it doesn’t happen regularly. Especially if the person still has their own teeth, because you will get all sorts of spots or blisters and things like that. You just want to prevent that.”*

(Carer, Interview 5)

Consistent with the finding that the unsuccessful provision of tooth brushing was often not considered a serious harm, some participants would not brush patients’ teeth involuntarily. At the same time, however, some caregivers would use restraints to change an individual’s incontinence material, indicating they prioritized anal hygiene over oral hygiene.

Interviewer: *“If we look at other care, they [individuals with dementia] don’t shower or don’t remove incontinence material. Would you then use some form of coercion if it really doesn’t work?”*

Participant: *“Yes, you would have to, because otherwise their buttocks would get damaged.”*

(Carer, Interview 1)

Moreover, when a patient showed resistance toward tooth brushing, carers were often inclined to discontinue oral care and did not attempt to provide it at a different moment. In other words, not all alternative options to brush someone’s teeth voluntarily were exhausted. Some participants explained this was due to organizational skills, time, and lack of awareness about the importance of daily oral care. Others explained that dental ADL-care (e.g., tooth brushing, cleaning prosthesis) is not given the same priority as non-dental ADL-care, such as showering. To illustrate, a geriatric specialist reported the following about resistance toward showering:

*“…we all look at what is going on: why someone doesn’t want to be put under a shower. That could be because they are afraid of water. So, we will see if we can do it with washcloths. In any case, alternatives are considered, but when it comes to oral care, if someone doesn’t want to, [oral care is] stop[ped].”*

(Geriatric specialist, Interview 2)

#### 3.1.4. Oral Care Provision

The identified barriers and facilitators concerning oral care provision were divided into four sub-themes: (A) psychology caregivers, (B) attitudes concerning involuntary oral care, (C) ethical and legal considerations, and (D) sedation.

(A) *Psychology of caregivers*

Focusing on the psychology of caregivers, someone’s own negative feelings toward oral care or dental visits could be vicariously projected onto the patient. This could complicate dental treatment, as it can increase patient resistance.

*“If we pick up the clients for dental treatment and someone from the care staff or the residential assistant walks with them, and they, start saying things like ‘Well, you don’t have to be afraid’ or ‘I hope the molars won’t be pulled’ or ‘Better you than me’, then we are looking like what are you doing?”*

(Dental Hygienist, Interview 2)

Another psychological factor was practical consistency. This could form a barrier as well as a facilitator. Some participants were disinclined to provide involuntary oral care due to their own aversion to receiving such care, whereas other participants did not understand why oral care was often forgotten, as they themselves would also want to receive it.

*“…well, then you have to put the toothbrush in the mouth of the patient. Yeah right, I’m really not going to do that, you wouldn’t want that for yourself either.”*

(Carer, Interview 1)

*“I can’t imagine why people don’t do that [tooth brushing] in evening care, because you wouldn’t go to bed without brushing your teeth yourself, would you?”*

(Nurse, Interview 1)

In addition, the importance assigned to someone’s own oral health could also influence their decision whether or not to provide involuntary oral care.

*“It also really depends on the care provider, of course. One care provider, you see them smile at you once, and you think, ‘Well, you never touched a toothbrush yourself, so how important do you think it is for yourself? So how important do you think it is for someone else?’”*

(Dentist, Interview 5)

Furthermore, some care providers preferred a patient’s family performing involuntary oral care because they find it to be a troubling experience. Also, the law does not apply to informal caregivers, and their involvement could help alleviate the workload of the nursing staff.

*“...and to be very honest, look, if the family does it with a bit of gentle coercion, so to speak, I find that less troubling than if we have to do it ourselves.”*

(Nurse, Interview 2)

(B) *Attitudes*

Looking at the second sub-theme, attitude concerning involuntary oral care was reported as influencing the decision whether to provide it. For instance, dentists had different opinions about indications for involuntary oral care provision. Some dentists would only consider a necessary extraction an indication for sedation, while others also considered a better prognosis of teeth a sufficient indication to make fillings.

*“When I provide involuntary care, I really only do extractions.”*

(Dentist, Interview 3)

*“I have sometimes chosen, in consultation with the doctor, to use Dormicum and fill as many teeth as possible so that they are easier to keep clean and the prognosis for the teeth is better, so they last longer.”*

(Dentist, Interview 2)

The attitude of a patient’s family could be a facilitator as well as a barrier to the provision of involuntary oral care. To illustrate how their attitude could be a barrier, the family could feel sorry for their relative if involuntary oral care were provided, and for that reason refuse to give consent for treatment.

*“…you also help someone get rid of an infection that can have a very negative impact on their health, but that is sometimes just difficult to explain to the family, especially if the family or caregivers themselves are not keen on the dentist. Then they say, ‘Oh, how sad, do I really have to put my mother through that?’”*

(Dentist, Interview 2)

Next to the attitude of dental professionals and family, the attitude of medical doctors also influenced treatment decisions. For example, some doctors were reported not to support sedation for involuntary oral care. Another example is a geriatric specialist who stated that if a patient has a life expectancy of one year, brushing their teeth is not necessary.

*“ …or the doctor who usually finds extractions fine, but when it can’t be done without sedation, they back out, at least my doctors do. They don’t dare to do that. That’s a bridge too far for them.”*

(Dentist, Interview 6)

Participant: *“I think, a set of teeth that deteriorates, well, that’s okay in a year.”*

Interviewer: *“So you’re saying that it doesn’t actually need to be brushed if someone has a year to live?”*

Participant: *“It doesn’t need to be, no.”*

(Geriatric specialist, Interview 1)

Finally, the attitude of carers and nurses also influenced the provision of involuntary oral care. For instance, some carers and nurses felt, just like family, sorry for the patient if involuntary oral care were provided. Also, some carers and nurses had a negative attitude toward dental professionals, which could influence their collaboration.

*“I do think that dental professionals are not very popular here, it indeed has a lot of negative influence on the ward or the housing units when they come here.”*

(Nurse, Interview 7)

(C) *Ethical and legal considerations*

Regarding the third sub-theme, participants described that their decision whether to provide involuntary oral care depended on multiple ethical and legal considerations. For instance, a participant explained that the average life expectancy of a newly admitted patient to the nursing home is between nine months and two years. With the patient’s life expectancy in mind, healthcare providers consider which treatments are necessary.

*“So, what are you going to do to those people who don’t want anything anymore in those two years? What are you going to do to them?”*

(Dentist, Interview 3)

Furthermore, the patient’s safety and the safety of others involved were also reasons for (not) providing involuntary oral care.

*“We must also think of our own safety. And if someone just starts hitting and kicking, then it’s simply, yes, that’s it.”*

(Carer, Interview 2)

Safety could also be a facilitator for providing involuntary oral care. For example, if a dental professional was performing a dental treatment with sharp instruments and the patient showed resistance while the instruments were still in their mouth, the dental professional might need to use involuntary care to prevent harm to the patient. Focusing on the safety of individuals around the patient, the behavior of the patient could be negatively affected due to oral pain, which might in turn affect the individuals around them. The patient could, for example, get aggressive toward others and cause them harm. Next to behavioral changes, the safety of other individuals could also be endangered due to contamination with bodily fluids:

*“…she left a trail of saliva and blood in the nursing home. At that moment, we thought something had to be done, because she was contaminating the nursing home and was also a danger to the other residents. […] I extracted teeth for her, and in the end, two weeks later, she had really improved a lot.”*

(Dentist, Interview 4)

Another consideration influencing decisions on whether to provide involuntary oral care was a patient’s dignity. Here is an example where dignity is a facilitator:

*“… in the light of dignity. Then I think, someone has always paid so much attention to it [their teeth]. Who are you, then, to decide, because that is essentially what you are doing, that someone no longer finds it important?”*

(Dental Hygienist, Interview 1)

However, the patient’s dignity could also be a barrier:

*“If you need two people to completely hold the head in place to fix something, that’s a struggle. Then I stop, and we discuss again. This is a bit dehumanizing.”*

(Dentist, Interview 6)

Furthermore, patient well-being could also be a reason to (not) administer oral care involuntarily. Their physical well-being could be endangered due to a health threat (e.g., pain, infection, difficulty with eating, or chance of suffocation due to mobile teeth):

*“…we had such a strong suspicion that something was going on in the mouth, something we couldn’t identify, which caused the person to lose weight, stop eating, become irritable, and so on. So, we thought, we need to look inside the mouth.”*

(Nurse, Interview 2)

In addition, a patient’s social well-being could be endangered due to, for example, severe halitosis:

*“… I’m talking about someone who had very bad breath. That becomes a burden for the entire department and the care staff. When they come by or sit at the table, everyone thinks ‘aaaargh,’ and then it becomes a social problem.”*

(Dentist, Interview 8)

Another reason for (not) providing involuntary oral care was the patient’s autonomy. This could play a role in two different ways. Naturally, the patient’s present autonomy could be a barrier, as multiple participants stated that the individual, despite their dementia, was still in charge, and that they would not use involuntary care if they showed resistance toward care.

*“We can’t suddenly start treating them like children. They are still in charge, so to speak, of their own bodies.”*

(Carer, Interview 2)

On the other hand, the autonomy a patient had prior to the dementia diagnosis, their past autonomy, was sometimes mentioned as a facilitator.

*“…you sometimes see that people have invested a lot in their teeth or they have spent a lot of energy, time, money, and so on, to maintain good teeth. And if you then handle it very lazily because you think, ‘Yes, someone shows resistance,’ or you stop at the slightest resistance, I think, ‘Is that really justifiable in light of their past autonomy?’”*

(Dental Hygienist, Interview 1)

A final ethical consideration was a patient’s biography.

*“A lady who refused all care and whose medical history stated that she had been abused at a young age. I don’t know exactly what happened, but it was something physical, which made her not want to be touched by anyone. […] I knew she still had a few teeth, at least, we thought so, but no one had ever looked inside her mouth. And we didn’t want to look either, because she had refused to be touched her entire life. So, we respected that.”*

(Dentist, Interview 4)

*“We take people’s life history into account. If someone has been in a concentration camp, for example, this becomes incredibly important, their autonomy and ability to do things for themselves. This can be so deeply ingrained. We actually had someone like that, where even basic daily activities were incredibly challenging because it meant entering their personal space. And when it comes to the mouth, that’s even more so…”*

(Geriatric specialist, Interview 3)

Regarding legal considerations, several participants believed legislation was a reason to discontinue daily oral care in cases of resistance. Potentially due to fear of legal ramifications, the law was reported to be used as an excuse to not provide oral care.

*“… they [carers] now think, Care and Coercion Act, oh, it’s not allowed, I won’t do it.”*

(Nurse, Interview 2)

All in all, several healthcare providers reported that there is no ethical or legal checklist for which cases they should provide involuntary oral care. In their choice to provide involuntary oral care, they considered a large variety of considerations and values.

*“How do we assess, what is someone’s life expectancy? What is the level of suffering if we don’t do it versus if we do? How do these factors relate to each other? Is what we are doing proportionate? I can’t provide a checklist for this in advance because it’s so individually determined.”*

(Geriatric Specialist, Interview 3)

*“Well, I think if a tooth were to be inflamed, and you would expect quite severe symptoms from that, it [involuntary care] is something worth considering. And I can’t just say one, two, three, ‘we’ll do it.’ Of course, there are multiple factors that are important in such a case.”*

(Nurse, Interview 5)

*“...then I’m willing to wedge my mirror in just a little bit to briefly check if there’s any acute issues, and then I accept it [care-resistant behavior] because these are often people who have a limited life expectancy anyway.”*

(Dentist, Interview 4)

(D) *Sedation*

Sedation, a form of involuntary care, can be used to provide oral care to individuals with dementia who show care-resistant behavior. A factor forming both a barrier and a facilitator was the existence of clear guidelines and protocols concerning sedation. Without clear guidelines and protocols, it is difficult to form a firm policy or ensure coordinated care within a team. The guidelines and protocols could lack specificity, and because of that lead to difficulties regarding the sedation of a patient. For example, one participant reported a case where the sedation was already administrated to a patient before the dentist was present and consequently had almost worn off before the treatment started:

*“I really like to be present when someone is going to be sedated because then you are much more involved and can treat at the right time. The last time it happened to me, they said they gave it at 12:30, and we were only called at 1:00, and by then the sedation had practically worn off.”*

(Dentist, Interview 1)

Another example of nonspecific guidelines and protocols was described by a dentist who encountered an unauthorized person administrating the sedative and monitoring for respiratory depression. This person was not trained to administer sedatives or how to check for respiratory depression. Lack of knowledge and protocols could lead to potential treatment complications.

*“... the person who was sitting next to him, supposedly monitoring his breathing, who had administered the midazolam [sedative], turned out to be a personal assistant, an intern, so not someone who was allowed to do that at all. […] I actually think that our protocols just need to be more watertight, so that you have well-documented responsibility for both the doctor and the nurses. So that they themselves have also seen on paper what their responsibilities are during the administration of midazolam.”*

(Dentist, Interview 7)

When the guidelines and protocols are clear, they could also serve as a facilitator. To illustrate, the protocol could consist of specific agreements concerning timing and method of sedation, and outline responsibilities for those involved (for example, the responsibility of monitoring for respiratory depression), the availability of instruments (such as pulse oximetry), and the agreements made for a worst-case scenario.

Furthermore, dentists found the presence of a doctor during sedation pleasant. Some dentists would not use sedation if the doctor was absent, and experienced barriers regarding the collaboration. To illustrate, a dentist said the following about a sedation appointment:

*“…I’ve arranged everything. And then, I go to check. No, there is no doctor at all. There is no doctor. Didn’t we agree that today? ‘No, well, but we often do little puffs in the nose’, so I say, ‘No, I don’t want that’.”*

(Dentist, Interview 3)

Dentists also experienced the presence of a patient’s loved one(s) as helpful as they often could calm the patient, and it also eased the loved one’s perception of the treatment’s severity. One dentist said the following about the help of a loved one after she gave the sedative:

*“I am not going to hug someone, but a daughter or a granddaughter will. So, they can really help them calm down, which I always find nice.”*

(Dentist, Interview 3)

In addition, some geriatric specialists lacked knowledge about sedation and its potential treatment complications.

*“…that lack of knowledge is also present in my profession, those who say: ‘give midazolam,’ they don’t know about the phenomenon of respiratory depression either.”*

(Geriatric specialist, Interview 2)

To increase the effectiveness of sedation, sedation for non-dental care could be combined with sedation for oral care. In this way, a dentist could benefit from the sedation episode by, for example, performing a dental examination of a patient who resisted care and is sedated for another reason.

*“…it’s not just about the mouth. Often, it’s also the ingrown toenail or if they need to be bathed daily, which also doesn’t work out. So, there is often much more going on, and they are frequently given midazolam for other reasons as well. I always try to combine it*.”

(Dentist, Interview 7)

A barrier to combining oral care with other care is uncertainty about responsibility. For instance, for dentists, it was not always clear who was responsible when they performed a dental examination after the sedative was administrated for other care:

*“If I know that they have taken that pill, I don’t like it because, what if they have problems? After they have been sedated, am I responsible at that moment? Is the care responsible at that moment? I think that is an important issue. Is it because I am looking into their mouth again that they become extra nervous and then have breathing problems? I don’t know, I find it quite difficult.”*

(Dentist, Interview 3)

## 4. Discussion

This paper reports the barriers and facilitators concerning involuntary oral care provision to older individuals with dementia who show care-resistant behavior. Most of the identified barriers and facilitators concerned daily oral care provision. An important result was that non-dental professionals often forgot oral care provision and prioritized non-dental ADL-care over dental ADL-care in case of care-resistant behavior. For instance, several care providers reported discussing resistance toward showering at an MDM and searching for alternative options to provide care voluntarily. However, when patients with dementia resisted oral care, several care providers did not mention this at the MDM. They would not search for alternatives to provide oral care voluntarily but instead discontinued oral care. This raises the question: what makes the provision of dental ADL-care different from non-dental ADL-care in case an individual with dementia shows care-resistant behavior?

A potential reason for deprioritizing dental ADL-care could be the reported lack of awareness of oral health problems and their consequences, which was also described by Hoben et al. [14] and Bellander et al. [17]. Bellander et al. [17] reported a lack of oral care teaching both in healthcare workers’ basic education and at their workplace [17]. Nurses and carers in the present study reported that their oral health education consisted solely of brushing each other’s teeth once. In addition, a geriatric specialist explained that oral care was discussed minimally during their education.

Another explanation for deprioritizing oral care could be the attitudes of non-dental professionals. For instance, a geriatric specialist stated that they would not brush the teeth of an older individual with dementia who had a life expectancy of one year. Consistent with this result are the results of Jonker et al. [13], who reported that only 9.7% of doctors would be willing to brush the teeth of an older individual with dementia who showed resistance [13].

In addition, some nurses and carers expressed a less positive attitude regarding (involuntary) oral care provision, which was also reported in multiple other studies [18,19]. Several nurses and carers experienced the presence of dental professionals in their workplace as a negative influence, likely due to challenges with regard to communication. Some participants explained that when dental professionals visited the nursing home, the primary feedback they received was criticism regarding the lack of toothbrushing. While this feedback was likely intended to be helpful, nurses and carers probably experienced it as discouraging. Instead, they expressed a desire for more support related to oral care from dental professionals. Supporting this outcome are two studies that also reported a lack of support by dental professionals as one of the most frequent barriers concerning oral care provision to nursing home residents [15,20].

Also, some caregivers had a negative attitude toward dental care in general, which they projected onto their patients. They felt sorry for their incapacitated patients for undergoing dental treatment, as they themselves would not want to receive dental treatment. For them, this justified the discontinuation of oral care in case of care-resistant behavior, instead of encouraging a search for voluntary alternatives to provide oral care.

A third possible explanation for deprioritizing oral care is the delayed consequences of not providing daily oral care and the lack of visibility of these negative consequences. Not providing daily oral care once will rarely cause immediate harm. However, consistently not providing it could result in serious harm over time [21,22]. This delayed impact could make it harder to recognize the importance of providing daily oral care. To illustrate, one carer indicated they would consider involuntary care to remove soiled incontinence material, likely because not removing this will cause direct visible (and odorous) harm. But at the same time, this carer reported that they were unwilling to brush their teeth involuntarily as they do not see the consequences. Furthermore, oral health problems remain behind closed lips and caregivers literally do not see the problem [23].

Finally, the fact that oral care was treated differently than non-dental ADL-care could also be due to logistical reasons. For instance, some nurses reported that because of a lack of manpower, time, materials, and budget, choices must be made, with oral care often being deprioritized. Similarly, Bellander et al. [17] explained that an increase in nurses’ daily workload required performing more nursing tasks, which led to poor motivation for nurses to provide oral health care [17]. Providing involuntary oral care is even more time-consuming than regular oral health care. For most care providers this will involve first attempting all alternative strategies to provide care voluntarily. The present study revealed that many carers lacked time and organizational opportunity to attempt providing oral care at a different time or in another way. Similarly, Gjellestad et al. found that when faced with resistance, nurses often lacked time to revisit the patient and attempt the task again [24]. Moreover, actually providing involuntary oral care, e.g., by sedating or restraining a patient, will take a lot of time and energy as well. And this may also expose carers as well as patients to emotional stress [11].

The present study found a lack of communication and reporting about unsuccessful tooth brushing, which is unfortunate as Willumsen et al. [9] reported that 52.4% of the patients with dementia resisted assistance with toothbrushing [9]. This indicates that a failure to provide daily oral care is a very common problem that should be discussed more during MDMs. Not providing daily oral care will lead to poor oral health, which can in turn influence an individual’s well-being and general health by causing pain or infections [8,21,22].

When all voluntary alternatives to provide oral care have been attempted and have failed, involuntary oral care could be considered an option. Whether a caregiver should provide involuntary care or discontinue care can often be a tough ethical dilemma. While some ethical considerations support involuntary oral care, others favor discontinuing care.

Moermans et al. [25] found that caregivers’ decisions related to providing involuntary care depended on their judgment, particularly whether safety outweighed autonomy [25]. Autonomy was also an important consideration for carers in the present study. Several participants reported that a patient’s autonomy was decisive in considerations about involuntary oral care. In other words, when a patient explicitly rejected oral care, that was sufficient for them to discontinue care. This outcome is supported by Weening-Verbree et al. [15], who reported that respecting patient autonomy led to regarding oral care as a second priority in ADL-care [15]. For some caregivers in their study, oral care was considered a service, not a part of primary care [15].

Hence, some caregivers, both in our study and in the study of Weening-Verbree et al. [15] prioritized autonomy over safety in the case of daily oral care provision. At the same time, however, several caregivers in our study did prioritize safety over autonomy. Especially in cases of pain or visible consequences of an oral health problem, such as weight loss or behavioral changes. This aligns with findings by Geddis-Regan et al. [26], who identified the presence of dental symptoms, such as pain, as the key factor in providing involuntary oral care [26].

Associated with patient autonomy, participants in the present study distinguished between a patient’s current autonomy and their past autonomy. While respecting a patient’s current resistance might point toward discontinuing care, their past attitude toward oral health might favor involuntary care. Furthermore, apart from safety and autonomy, participants also mentioned various other values that were relevant for dealing with the dilemma. They mentioned dignity or humanity, quality of life, life expectancy, well-being of fellow residents, well-being and safety of caregivers, prognosis, practical consistency, and patient biography.

Given this large variety of values influencing a decision to provide or not provide involuntary oral care, it is not surprising that participants reported that a judgment on whether involuntary oral care is justified cannot be made in some general manner. In other words, the presence of a reported facilitator does not automatically mean that involuntary oral care will be provided, just as the presence of a barrier does not automatically imply that involuntary care will not be provided.

Instead, ethical considerations often influenced decisions about involuntary oral care in a joint fashion. To illustrate, some dentists described choosing not to treat an infected tooth that poses a risk to a patient’s well-being because that would respect their present autonomy and because they had a short life expectancy. In addition, decisions about involuntary care were often based on the specific characteristics of the individual patient. For instance, some care providers reported not providing involuntary care to patients with a history of physical abuse, and others reported they rather provided involuntary oral care to individuals who would have consented to the treatment in the past. Interestingly, dentists turned out to disagree when it comes to decisions about involuntary oral care. While some dentists only use sedation to extract painful teeth, others also consider an increase in the prognosis of teeth a reason to use sedation. This indicates the complexity of the choice to administer involuntary oral care. Dentists may be assumed to have sufficient dental knowledge, and as the dentists expressing different opinions had taken the same geriatric dentistry training, their attitude is determined by considerations other than merely clinical expertise. Accordingly, perceptions of which involuntary measures count as ‘proportional’ in a specific oral situation also vary widely, as these, too, are influenced by a caregiver’s personal values.

Apart from considerations influencing the decision to provide involuntary care or not, its actual provision also had barriers and facilitators. One interesting result is that caregivers find it helpful if the family of the patient assists in performing involuntary care, for example, by holding the patient’s hands. Similarly, nurses in the study by Moermans et al. [25] experienced the help of family members during involuntary treatment as very supportive, as they assisted with tasks such as clamping the patient’s arms during hygienic care or by distracting them [25].

### Strengths and Weaknesses

Multiple studies identified care-resistant behavior as a barrier to providing (oral) care, but they often failed to explore what follows once this behavior occurs. This highlights a gap in the current literature concerning involuntary oral care provision. The current study fills this gap, by finding more values underlying care providers’ decisions to provide or not provide involuntary oral care. Another key strength of the present study is its qualitative approach, which made it possible to identify actual barriers and facilitators concerning involuntary oral care provision from the perspective of care providers. Additionally, the study benefits from a large variation in healthcare providers.

A limitation of our study is that some identified barriers and facilitators given by participants are rather speculative. For example, some dentists suggested that the (low) importance carers attach to their own oral health could well influence their reluctance to provide involuntary oral care. Similarly, several non-dental professionals reported that a clinical lesson provided by a dentist could possibly be helpful for providing involuntary oral care. However, barriers and facilitators like these remain hypothetical rather than verified facts.

Another limitation of the present study is its primary focus on the Netherlands, where a specific Dutch law regulates involuntary (oral) care. Therefore, the transferability of the results may be limited. Follow-up studies should be conducted in other countries, as they have different laws, and care providers potentially have different barriers and facilitators concerning involuntary (oral) care provision to older individuals with dementia who show care-resistant behavior.

## 5. Conclusions

Our study shed more light on the barriers and facilitators regarding involuntary oral care provision to older individuals with dementia. Barriers and facilitators were experienced concerning communication (between dental and non-dental caregivers, and among non-dental care providers themselves), logistics (materials, transportation, and staff and time), knowledge (training, awareness of oral health problems, and assessment of severity of oral health problems), and oral care provision (psychology caregivers, attitude concerning involuntary oral care, ethical and legal considerations, and sedation).

### Recommendations

- Designate nurses and train them with specific expertise in oral health to help monitor oral health in the workplace and improve communication between dental and non-dental care providers.
- Involve dental care professionals in MDMs.
- Organize training for care providers regarding (1) managing care-resistant behavior and finding voluntary ways to provide care, (2) the importance of good oral health and tips/tricks for providing oral care, and (3) more training on the law to prevent it from being used as an excuse.
- Make sure that a shift in attitude is discussed as this is necessary to make sure that oral care is treated the same as non-dental ADL-care.
- Provide or design clear guidelines and protocols for sedation and daily oral care provision (for instance, after how many failed attempts to provide daily oral care should it be considered a problem?).
- Perform more research on providing involuntary oral care, including practical examples where the decision was made to provide involuntary oral care and the reasoning behind it.

## Figures and Tables

**Table 1:** Summary of semi-structured interview guide [10].

1. What is your work function? How long have you been doing this job?
2. What does a working day look like for you?
3. In what way are you involved in the oral care of residents?
4. Do you ever deal with residents who show care-resistant behavior toward oral care? What do you do in such cases?
5. What are your experiences with providing involuntary oral care? Can you give examples? Do you encounter certain issues (barriers) concerning involuntary oral care? What would make it smoother for you to perform involuntary oral care (facilitators)?
6. What do you consider reasons to provide involuntary oral care as a last resort? What is your opinion concerning involuntary tooth brushing?
7. What do you consider involuntary care? Can you give examples?
8. Does the Care and Coercion Act play a role for you in providing involuntary oral care? How so?
9. What is your age, what is your gender, and in what province do you work?

**Table 2:** Summary of participants’ characteristics.

Occupation	Gender	Age
Dentist	Male	79
Dentist	Female	43
Dentist	Female	58
Dentist	Male	60
Dentist	Female	34
Dentist	Female	47
Dentist	Female	41
Dentist	Female	65
Dental hygienist	Female	40
Dental hygienist	Female	35
Dental hygienist	Female	32
Carer	Female	43
Carer	Female	36
Carer	Female	57
Carer	Female	51
Carer	Female	37
Carer	Female	50
Nurse	Female	58
Nurse	Female	30
Nurse	Female	33
Nurse	Female	41
Nurse	Female	59
Nurse	Female	55
Nurse	Female	27
Geriatric specialist	Male	64
Geriatric specialist	Male	66
Geriatric specialist	Female	57
Dementia case manager	Female	32
Dementia case manager	Female	31
General practitioner	Female	55
General practitioner	Female	53
General practitioner	Female	46

## Data Availability

The data presented in this study are available on request from the corresponding author. Please note that the data are available only in Dutch.
